# General and Specific Stress Factors as Potential Predictors of Work Ability Among Pre-Hospital Emergency Medical Personnel

**DOI:** 10.3390/healthcare14131854

**Published:** 2026-06-25

**Authors:** Nikola Bajan, Marija Raguž Vinković, Mario Vukušić, Antun Bajan, Dubravka Matijašić-Bodalec, Ana Mehičić, Petra Mamić, Krešimir Šolić

**Affiliations:** 1 Faculty of Medicine, Josip Juraj Strossmayer University of Osijek, Josipa Huttlera 4, 31000 Osijek, Croatia; nbajan@mefos.hr (N.B.); kresimir@mefos.hr (K.Š.); 2 Faculty of Dental Medicine and Health, Josip Juraj Strossmayer University of Osijek, Crkvena 10, 31000 Osijek, Croatia; 1mariovukusic@gmail.com (M.V.); antun.bajan@fdmz.hr (A.B.); 3 Mlinarska School of Nursing, Mlinarska Cesta 34, 10000 Zagreb, Croatia; dubmatij@gmail.com; 4 Clinical Hospital Center Osijek, Josipa Huttlera 4, 31000 Osijek, Croatia; mehicicana1@gmail.com; 5 Croatian Institute of Public Health, Rockefellerova Ulica 7, 10000 Zagreb, Croatia; petra.bodalec@gmail.com; 6 Faculty of Tourism and Rural Development in Požega, Josip Juraj Strossmayer University of Osijek, Vukovarska Ulica 17, 34000 Požega, Croatia

**Keywords:** emergency medicine, workplace, occupational stress, job satisfaction, healthcare workers, Croatia

## Abstract

**Background/Objectives**: Retention of healthcare professionals in the workforce, their employment, and the improvement of working conditions largely depend on identifying the factors that influence their departure and their health. The study was conducted during the period from January to June 2021. This study aimed to examine the association between specific work-related stressors and work ability. The initial hypothesis was that general and specific occupational stressors negatively associate with work ability among healthcare professionals in emergency medical intervention teams. **Methods**: The study was designed as a cross-sectional comparative study. It was conducted among nurses and physicians in pre-hospital emergency medical services, employed full-time in intervention teams, while the control group consisted of employees from dispatch and call-receiving units. The study was conducted on the 840 participants, representing 43.3% of all healthcare professionals employed in pre-hospital emergency medical services in the Republic of Croatia. In addition to questions on participants’ personal characteristics, the following instruments were used: 1. a validated Questionnaire on Workplace Stressors among hospital healthcare professionals; and 2. the international standardized Work Ability Index (WAI) questionnaire for assessing work ability. Participants completed the questionnaires in paper form. **Results**: On average, the participants demonstrated lower levels of stress compared to reference values, both for overall stress and for individual stress factors, while their work ability, assessed using the Work Ability Index (WAI), ranged from very good to excellent. The control group showed higher levels of stress across all factors and lower work ability. However, the control group was older on average, generally had lower levels of education, and consisted more often of women—personal characteristics that may influence the examined variables. Lower stress levels and better work ability were associated with job satisfaction, ambition, and the fact that participants were working in their desired profession. Frequent sick leave (absenteeism) was highly correlating with both higher stress levels and poorer work ability. **Conclusions**: Greater job satisfaction and higher motivation have a positive impact on stress levels and employees’ work ability. The study results can serve as a starting point for institutional management in designing feasible decisions aimed at improving satisfaction, health, the work environment, and the work ability of emergency medical service personnel, as well as making these institutions more attractive for recruitment and retention of employees both in their positions and within the profession.

## 1. Introduction

Healthcare workers represent the largest workforce in the world, with nurses comprising the largest proportion. In recent years, a shortage of healthcare professionals has emerged, becoming a global problem. Retaining nurses in the profession, recruiting new staff, and improving working conditions are strategic goals that largely depend on identifying factors influencing their decision to leave the profession. Numerous international studies indicate an increasing number of nurses willing to change jobs. This phenomenon represents a pressing issue for healthcare systems in many countries [1,2], particularly in less developed European Union countries [3,4,5].

The shortage of healthcare workers is also a challenge faced by the Republic of Croatia. Existing working conditions are identified as a leading cause of this problem [6,7]. Working conditions, work organization, the development of healthcare technologies, scientific advancements, professional knowledge, and diagnostic and therapeutic capabilities significantly increase the psychophysical demands placed on healthcare workers [8].

Numerous studies have shown that an empowering work environment can increase nurses’ job satisfaction by providing a sense of support and encouraging more efficient task performance. A Portuguese study states that structural empowerment of nurses directly increases job satisfaction and indirectly reduces burnout. It includes systems, policies, and resource allocation at the organizational level and can directly influence the work environment and job satisfaction. Psychological empowerment refers to a subjective sense of control, autonomy, meaning, and competence. It is linked to an internal sense that fosters hope for making significant changes in both professional and personal life [9]. Over the past century, the world has experienced four major pandemics [10].

The term stress originates from the medieval English word “stress” (meaning hardship, adversity, or constraint), and was used as early as the 14th century to describe suffering, difficulties, misfortune, or sorrow [11]. Today, stress describes a state in which the body perceives a threat to its integrity. The physiological model of stress was established in the 1930s by Hans Selye, who demonstrated that a wide range of stimuli can produce the same physiological changes, referring to them as “stressors.” Stressors are stimuli that cause stress and reflect specific situations or events. A lack of recovery periods between stressors leads to chronic stress, which is a common cause of more serious health problems [12]. In addition to stressors, environmental factors and individual sensitivity interact to produce responses that may lead to illness. Lifestyle, age, and personality determine individual sensitivity, while friends, work environment, surroundings, and family are part of external factors [13]. Numerous studies have shown that approximately 75–90% of various diseases are associated with stress [14]. Stress is closely linked to impaired brain function, including memory, cognition, and learning [15]; it affects immune system function through neuroendocrine regulation [16]; it has harmful effects on the cardiovascular system, increasing the risk of thrombosis, atherosclerosis, coronary vasoconstriction, cardiac arrhythmias, and myocardial infarction [17]; and it is associated with gastrointestinal complications affecting appetite and normal digestive function.

Maintaining and promoting work ability is of great societal importance [18]. In the past, work ability primarily required physical fitness and capacity. Today, physical work increasingly includes mental work, along with newer approaches such as teamwork. These changes have led to a shift in the definition of work ability, which is now considered a multidimensional concept aimed at prolonging working life and identifying factors that may affect it as early as possible [19]. Work ability can be defined as an individual’s capacity to perform a job, taking into account job demands as well as health and psychological capabilities [20]. A person is considered capable of working when they can balance job demands with their personal resources [13]. Various factors influence work ability, including working conditions, job competence, age, family conditions, and living circumstances. Some jobs require increased physical and psychological effort, while others involve exposure to biological, physical, and toxic hazards or risk of injury [20]. In addition to the individual, it is essential to consider the type of work and the work environment [21]. This holistic model was developed in the early 1980s at the Finnish Institute of Occupational Health. As part of a research project, the Work Ability Index (WAI) was created as a tool for assessing employees’ work ability [11,22,23].

Following its accession to the European Union, Croatia has faced significant and rapid challenges related to its healthcare system, including working conditions, labor market changes, education of healthcare professionals, job systematization, and other reforms aimed at making the system more functional and flexible. Emotional trauma caused by pandemics, natural disasters, war conflicts, and social crises leads to significant stress-related disturbances. Croatia has additionally been burdened by war on its territory and two major earthquakes, posing serious challenges to mental health [24]. These crisis situations have highlighted both strengths and weaknesses within the healthcare system. The COVID-19 pandemic posed a particular challenge, especially for pre-hospital emergency medical services, which are always the first point of contact with patients. Pre-hospital emergency medical services (EMS) in Croatia are organized at the county level (in 21 Croatian counties). Each county institute has its central medical dispatch unit and field teams. Pre-hospital EMS interventions provide EMS ground teams, EMS maritime teams and EMS helicopter teams. The number of emergency teams is determined by the national emergency services network established by the Ministry of Health. These teams include Team 1, composed of a physician, a nurse, and a driver; Team 2, typically staffed by a specialized emergency nurse; non-emergency medical transport units, consisting of a nurse and a driver; and dispatch units, usually staffed by nurses, with a physician present in some larger counties. The latest legal document detailing the structure and operation of emergency medical services and ambulance transport in Croatia has been in effect since 2024. This is the pre-hospital segment of emergency medicine in Croatia, which is separate from hospital emergency services. The pre-hospital emergency professionals demonstrated knowledge, adaptability, and willingness to help, but also their vulnerability in terms of illness and absenteeism. This resulted in staff shortages, placing additional pressure on those able to work, who faced difficult conditions, multiple shifts, and increased workload, particularly due to a higher number of emergency calls. Work in emergency medicine is inherently stressful, largely due to exposure to unexpected tragedies, time constraints, multiple patient demands, administrative burdens, and legal regulations [25,26].

A pilot study was conducted between May and July 2024 in two counties in the Republic of Croatia. The study showed that 92.8% of participants had good or excellent work ability. Furthermore, lower work ability was recorded among dispatchers and nursing staff in medical transport services (median 38.8) compared with Emergency Medical Team 1 (median 43.0) and Emergency Medical Team 2 (median 44.5). A significant association was found between work ability and the nursing practice environment, job satisfaction, and belief in future reforms [27].

Based on the above, the aim of this study was to examine the relationship between specific workplace stressors and work ability. The initial hypothesis was that general and specific work-related stressors negatively associate with the work ability among healthcare professionals working in emergency medical service intervention teams.

## 2. Materials and Methods

The study was designed as a cross-sectional comparative study [28]. The study was conducted at the national level in the Republic of Croatia, including 19 county Institutes of Emergency Medicine and two teaching institutes of emergency medicine in Istria County and the City of Zagreb. Participants included physicians and nurses/medical technicians employed in pre-hospital emergency medical services, regardless of their level of education or workplace assignment (Team I, Team II, or the dispatch/communication unit), who met the following inclusion criteria: full-time employment in an emergency medical service, voluntary participation in the study, and proficiency in the Croatian language.

Prior to conducting the study, the minimum required sample size was determined to be at least 330 participants, based on the requirements for statistical analysis. With a statistical power of 80% and a medium effect size, a minimum of 200 participants is required for a one-way analysis of variance (ANOVA) with five groups, while a minimum of 282 participants is required for Pearson’s correlation test (G*Power 3). The total number of healthcare workers in emergency medical service institutes is defined by the Emergency Medical Services Network, established by the Minister of Health upon the proposal of the Croatian Institute of Emergency Medicine, and includes 1940 individuals who meet the inclusion criteria [29]. Due to the expected high level of participation and response rate, the anticipated sample size of 840 participants exceeded the minimum required 330 (with an additional 10% to account for potential data loss). Of the 900 questionnaires distributed to the Emergency Medical Service Institutes, 60 were excluded from the study due to incomplete responses.

Participants were divided into two groups based on their job roles, workplace, and working conditions: a case group and a control group. The case group consisted of healthcare professionals working in intervention teams (Team I and Team II). The exclusion criterion for this group was employment in the dispatch/communication unit. The control group consisted of healthcare professionals working in the dispatch/communication unit, who were not part of intervention teams and were not exposed to the COVID-19 virus.

The research instrument consisted of two questionnaires and 20 open-ended questions:

1. Personal characteristics questionnaire: This included questions on gender, age, highest level of education, profession and job tasks, alignment of job with education level, studying alongside work (at a university or polytechnic), permanent employment status, workplace, working hours, total years of work experience, years at the current position, weekly overtime hours, commuting time (in hours), job satisfaction, perception of work life, working conditions, causes of sick leave, most common cause of sick leave, and exposure to occupational health risks.
2. Workplace Stressors Questionnaire for Hospital Healthcare Workers: With permission from the author Milan Milošević, this standardized questionnaire was used to identify and assess workplace stress factors. It includes 37 stressors grouped into six factors. Participants rated each stressor on a Likert scale from 1 (not stressful at all) to 5 (extremely stressful). Higher scores indicate a higher perceived level of stress. Scores above 60 suggest that a particular factor is considered stressful [30]. Factor analysis extracted six factors of relatively high reliability of the internal consistency type (all Cronbach α values were greater than 0.7).
  The stressors were grouped into six factors:
  ○ (F1) Workplace organization and financial issues (10 items): Inadequate salary, insufficient material resources, inadequate workspace, limited promotion opportunities, poor communication with superiors, insufficient staff, poor work organization, daily unforeseen situations, administrative tasks, work overload.
  ○ (F2) Public criticism and lawsuits (7 items): Threat of legal action, unrealistic patient expectations, inappropriate public criticism, misinformation of patients, conflicts with patients, inability to separate professional and private life, 24-h responsibility.
  ○ (F3) Workplace hazards and risks (6 items): Fear of ionizing radiation, fear of inhalational anesthetics, fear of infection, exposure to cytostatics, risk of injury from sharp objects, dealing with incurable patients.
  ○ (F4) Conflicts and communication at work (4 items): Conflicts with colleagues, conflicts with other staff, poor communication with colleagues, conflicts with superiors.
  ○ (F5) Shift work (4 items): night shifts, shift work, overtime work, 24-h duty shifts.
  ○ (F6) Professional and intellectual demands (6 items): Introduction of new technologies, information overload, lack of continuous education, time pressure, limited access to literature, time constraints for patient examinations.
3. Work Ability Index Questionnaire (WAI): Among widely used instruments, the Work Ability Index (WAI) is a valid self-assessment tool developed by the Finnish Institute of Occupational Health. It is used to assess work ability in relation to job demands. The questionnaire has been translated into Croatian and validated through the scientific research project “Occupational Health and Healthy Environment” (No. 1080316-0300) of the Ministry of Science, Education and Sports of the Republic of Croatia [12].

According to the Finnish Institute of Occupational Health, work ability is calculated by summing scores from seven dimensions included in the WAI. The questionnaire uses a Likert scale. The maximum score is 49 (indicating excellent work ability), while the minimum score is 7 (indicating very poor work ability). The internal consistency of the questionnaire was assessed using Cronbach’s alpha coefficient, and in our research a satisfactory result of α = 0.92 was obtained, indicating acceptable reliability. Based on the total score, work ability is classified into four categories:

1. Poor (7–27)—restoration of work ability required;
2. Moderate (28–36)—improvement needed and causes of low scores should be considered;
3. Good (37–43)—maintenance and future improvement recommended;
4. Excellent (44–49)—excellent work ability; maintenance of current state recommended [11].

Standard statistical methods were used: the Shapiro–Wilk test for testing normality, chi-square test for categorical variables, and nonparametric Mann–Whitney and Kruskal–Wallis tests with Conover post hoc analysis. Associations between numerical variables were assessed using Spearman’s correlation test, along with a linear regression model and coefficient of determination. Data were analyzed using MedCalc software (version 19.5.1, MedCalc Software Ltd., Ostend, Belgium), with a significance level set at α = 0.05. All *p*-values were two-tailed.

The study was conducted after obtaining approval from the Ethics Committees of the respective institutes and the Faculty of Medicine Osijek at the University of Josip Juraj Strossmayer in Osijek, during the period from January to June 2021 (Class: 602-04/21-08/07; Reg. No.: 2158-61-07-21-05). Participants were informed about the purpose of the study and provided informed consent. The principles of the Declaration of Helsinki were respected. Participants did not receive any incentives for participation. Prior to the study, written permission was obtained from the authors of both the Workplace Stressors Questionnaire and the Work Ability Index Questionnaire.

## 3. Results

A total of 840 participants were included in the study, all of whom completed the questionnaires and thus took part in the research. This represents slightly less than half of the total population, specifically 43.3% of all employees from the 19 county institutes and two teaching institutes of emergency medicine. The median age of the participants was 33 years, with an interquartile range of 27 to 44 years and an overall range from 20 to 64 years. A significantly higher proportion of participants (chi-square test, *p* < 0.001) were aged between 26 and 35 years, and more than half had a secondary level of education (chi-square test, *p* < 0.001). The sample included an approximately equal number of participants of both sexes (Table 1).

Slightly more than half of the respondents (Chi-square test, *p* < 0.001) are nurses and are not studying while working. The vast majority of respondents (Chi-square test, *p* < 0.001) are employed in positions consistent with their level of education and have permanent employment contracts (Table 2).

The majority of respondents (Chi-square test, *p* < 0.001) reported being fairly satisfied with their job, with only 4.5% expressing dissatisfaction. Furthermore, slightly more than two-thirds of respondents consider their work to be challenging, interesting, and meaningful. Slightly less than two-thirds believe that working conditions are generally not harmful to their health, although unpredictable circumstances may occur. A significant majority of respondents (Chi-square test, *p* < 0.001) had not taken sick leave, and this proportion increases when considering the period of the past 3 to 6 months. Approximately equal proportions of respondents, around one quarter each, reported that their job was occasionally the cause of sick leave, while a similar proportion stated that sick leave was due to other reasons rather than work (Table 3).

Only 2.4% of respondents, according to the WAI method, have poor work ability, while the median total score is 42 points (IQR from 37 to 46 points), which is very close to the lower threshold for the “excellent” category at 44 points. There is a weak but statistically significant negative correlation (Spearman’s correlation test, *p* < 0.001) between certain stress factors and work ability scores, indicating that increased stress negatively affects the respondents’ work ability (Table 4).

The coefficient of determination (rho^2^) for the overall perceived stress is 13.69, indicating that 13.7% of the total variance in work ability, as assessed by the WAI method, can be explained by the overall perception of workplace stress. The results of the analysis show that male respondents have significantly higher work ability (Mann–Whitney test, *p* = 0.002) and, at the same time, significantly lower overall perceived stress (Mann–Whitney test, *p* = 0.03) compared to female respondents (Table 5).

The length of service at the current workplace has a negative effect on work ability (Kruskal–Wallis test, *p* < 0.001). In contrast, respondents who are studying or planning to study have significantly higher work ability (Kruskal–Wallis test, *p* < 0.001). Similarly, employees on fixed-term contracts also demonstrate significantly higher work ability (Kruskal–Wallis test, *p* < 0.001), suggesting that fixed-term employment may be a positively associated with the work ability. Overtime work, as well as differences in occupation and job tasks, do not have a significant impact on the respondents’ work ability (Table 6).

The dispatch unit is exposed to significantly higher levels of stress (Mann–Whitney test, *p* < 0.001) and has significantly lower work ability (Mann–Whitney test, *p* < 0.001) (Table 7).

Job satisfaction is also a potential factors associated with the respondents’ work ability, with significant differences observed between all response categories (post hoc Conover test, *p* < 0.05). In other words, higher job satisfaction is associated with significantly greater work ability (Kruskal–Wallis test, *p* < 0.001). Similar results were found for other examined parameters related to job satisfaction. There is a significant association (Kruskal–Wallis test, *p* < 0.001) between higher work ability and perceiving work as challenging, while lower work ability is associated with potentially health-threatening working conditions and a greater perceived exposure to health burdens in the past 3 to 6 months (Table 8).

Work and occupational injuries as frequent reasons for sick leave are reported by respondents with significantly the lowest work ability (Kruskal–Wallis test, *p* < 0.001), whereas respondents with significantly higher work ability (Kruskal–Wallis test, *p* < 0.001) indicate that their job is precisely the one they want to do. Work ability decreases linearly with total length of service, according to the regression equation: y = 43.6 − 0.17x (SE = 0.002, *p* < 0.001) with the coefficient of determination R^2^ = 0.129, meaning that 12.9% of the variance in work ability can be explained by total length of service according to the regression model. Therefore, total length of service is a potential negative factor of respondents’ work ability. Overall, higher job satisfaction and greater motivation among respondents were shown to be potentially associated with the lower perceived stress and higher work ability. Sick leave is also associated with stress and work ability: respondents who have not taken sick leave exhibit significantly lower stress levels (Kruskal–Wallis test, *p* < 0.001) and significantly higher work ability scores (Kruskal–Wallis test, *p* < 0.001).

## 4. Discussion

The study confirmed the negative impact of general and specific work-related stressors on the work ability of healthcare professionals in emergency medical service intervention teams. Since the study included 43.3% all healthcare workers in intervention teams from all emergency medical institutes in the Republic of Croatia, the results provide an approximate picture at the national level. The research was conducted during the COVID-19 pandemic with a defined control group, thereby also examining the negative impact of the pandemic on the participants.

The results of this study, as expected, showed that the average healthcare professional in emergency intervention teams is, in fact, not under stress and has very good work ability. Emergency workers are driven by adrenaline, perform a job they love, and accept it as a calling, especially when working with emergency patients. Furthermore, slightly more than two-thirds of respondents consider their job challenging, interesting, and meaningful, while slightly less than two-thirds believe that working conditions are generally not harmful to their health, despite the possibility of unpredictable circumstances. Overall, for 83.0% of respondents, this job is exactly what they want to do or very close to it, and most respondents have not felt increased exposure to health-related burdens in their workplace over the past 3 to 6 months. Emergency workers are generally motivated and satisfied with their jobs, which is supported by the results of this study, showing that higher job satisfaction and greater motivation are potentially associated with lower levels of perceived work-related stress and, at the same time, higher levels of work ability among employees. The majority of respondents reported being fairly satisfied with their jobs, with only 4.5% expressing dissatisfaction. The results of this study are encouraging and leave room for further improvement, especially considering that workforce productivity and quality largely depend on morale, which is directly proportional to job satisfaction. This is particularly significant given that the findings were obtained during the COVID-19 pandemic—a period marked by a heightened risk of increased stress levels among emergency medical personnel who were on the front lines [31,32]. For this reason, emergency medical service administrations around the world initiated additional processes based on international standards to address the emerging crisis, while taking into account staff as key stakeholders in the implementation of all processes. Their satisfaction was therefore crucial for achieving the desired outcomes through these processes [33,34].

Using the validated Workplace Stressors Questionnaire [30], the overall perception of stress as well as stress related to individual factors—i.e., stressors—was examined. The results showed that the perceived level of stress among employees was well below the reference value, indicating a low level of stress both overall and across all measured factors. Although all stressors were rated very low, the factor related to public criticism and lawsuits received a slightly higher rating. The results of this study are consistent with research indicating that workload can be defined as a balance between job-related stress and an individual’s response to those demands [35]. Work-related stress may have either positive or negative short-term effects. In the long term, if negative stress is not adequately managed, work-related stressors can affect emotional well-being and are associated with depression, anxiety disorders, and cardiovascular diseases [36,37]. There are numerous models of stress; according to Rohmert and Rutenfranz, who describe the relationship between the work situation and its effects on the individual, stress can be considered largely value-neutral, although it varies from person to person [38]. This also implies that factors such as social/family background and personality influence how stress is experienced.

The internationally recognized Work Ability Index (WAI) questionnaire, translated into and validated in the Croatian language [10], was used to assess the work ability of emergency medical personnel. Only about every 40th employee was rated as having poor work ability, while the average work ability score for all employees, according to the WAI method, ranged between very good and excellent. Every 40th employee falls into the 2.4% of respondents, according to the WAI method, who have poor work ability. According to the literature, this group consequently has a high likelihood of transferring to another job, retiring, or leaving their position altogether if preventive measures are not taken—such as activities aimed at improving working conditions or enhancing employee health. For respondents (17.5%) who rate their work ability as good, measures should be implemented to further improve their work ability. In order to align workers’ capacities with job demands, individual, organizational, or societal measures are needed to maintain a level of work ability that may exceed what the job itself supports. The results of this study contrast with findings from international research reported in the relevant literature. Projections indicate a continuously growing shortage of employees [39,40]. Among paramedics in the United States, the annual turnover rate is nearly 10% [41,42]. In Israel, Dopelt et al. report a turnover rate among paramedics of 42% after two years of work and over 90% after 10 years [43]. A recent study from Germany showed similarly alarming figures: a total of 54% of paramedics considered leaving emergency medical services within one year, and 46% reported being dissatisfied with their job. It was also observed that paramedics have relatively lower levels of job satisfaction and higher levels of depression [44] and burnout [45]. By examining the relationship between perceived stress levels and work ability ratings, a weak but statistically significant negative correlation was confirmed between certain stress factors and work ability scores. As expected, the overall perception of workplace stress negatively affects employees’ work ability.

The results of the study confirm that female participants tend to be more sensitive to the perception of stress. Male employees demonstrate significantly higher work ability and, at the same time, a significantly lower overall perception of stress compared to female respondents. It appears that older participants are less resistant to stress factors, with middle-aged and the oldest employees experiencing higher levels of stress. Work ability also declines significantly with age. Although the overall perception of stress does not differ significantly between educational groups, the lowest average levels of stress across all measured factors were observed among respondents with the highest level of education. It is assumed that employees with higher educational attainment cope better with stress. Work ability, however, does not depend on the level of education. It seems that a higher level of professional qualification has a positive effect on health, despite being associated with greater responsibility [46]. Age, and therefore work experience, do not necessarily protect against stress. Results that take gender into account are not consistent [46]. Men have shown greater emotional exhaustion and depersonalization than women, but women with high levels of alexithymia have demonstrated higher levels of burnout [47]. Unfortunately, studies are not directly comparable, as a wide variety of questionnaires have been used. It is also unclear whether job titles are equivalent across countries. Care was taken to refer to typical job titles and to describe emergency medical services accurately. The level of evidence in these studies is relatively low, which may be due to the fact that cross-sectional studies generally provide limited evidence. It appears that age and work experience do not necessarily protect against psychological stress and its consequences [48]. This may be because the risk of exposure to traumatic events and acts of violence increases with years of service in emergency and rescue professions.

Respondents in the control group, consisting of healthcare professionals from the Medical Dispatch Unit (PDJ), reported significantly higher stress levels across all factors and had significantly lower work ability. However, the control group respondents were also significantly older, had lower levels of education, and were more often female compared to the case group. This may indicate that the higher stress levels and lower work ability are at least partly due to the personal characteristics of the respondents, rather than solely the nature of working in the dispatch unit. Additionally, working in the PDJ was identified as a potential predictor of higher levels of occupational stress, and the results showed that increased stress has a relatively weak but statistically significant negative impact on work ability. From this, it can be inferred that PDJ teams experience a more stressful work environment, even though they are not in direct contact with the COVID-19 virus. It is likely that the pandemic further increases stress among PDJ teams, as they are required to make decisions and perform triage, whereas intervention teams are generally better prepared—both psychologically and in terms of equipment—and have a clearer expectation of what awaits them when working directly with patients, including potential exposure to the virus.

It can be concluded that the obtained results indicate low levels of stress, high levels of job satisfaction, and very good to excellent work ability, regardless of the workplace. These findings are contrary to most results reported in international studies. Typically, work in emergency services is associated with additional challenges, such as dealing with unexpected tragedies, time pressure, working in highly complex environments, patient demands, and administrative regulations. The COVID-19 pandemic has further intensified stressful situations, including risks related to personal safety, the possibility of transmitting infection to family members, and numerous controversies surrounding epidemiological measures. It is well established that prolonged exposure to workplace stress has a negative impact on the psychological health of healthcare workers, which can indirectly affect patient care as well. Although healthcare workers in emergency medical services in the Republic of Croatia faced all these challenges during the COVID-19 pandemic, it appears that they did not leave a significant impact on their psychological state. Nevertheless, it should be kept in mind that, despite the currently positive results, emergency medical personnel remain continuously exposed to various stressors, and these favorable outcomes should be maintained or even improved. Achieving this goal requires additional efforts from employers, particularly in developing clear operational protocols that can help reduce stress and increase efficiency, thereby enhancing job satisfaction. Furthermore, providing continuous emotional and psychological support to healthcare workers fosters a sense of care for their well-being. It is also important to consider that improving the financial situation of healthcare workers contributes to greater job satisfaction. Ongoing education on stress-coping strategies can play a crucial role in empowering individuals to better manage stressful situations.

In conclusion, the working conditions and challenges faced by healthcare professionals in emergency services are very similar worldwide. Therefore, establishing unified global guidelines for coping with stress could be highly beneficial in preventing the negative consequences of exposure to stressful situations among emergency healthcare workers. The results of this study could also serve as a starting point for further research on stressors and factors influencing employee satisfaction, as well as for the development of preventive programs and response algorithms for potential future pandemics or other crisis situations—not only in the Republic of Croatia but also on a global level, regardless of the specific model or organization of emergency medical services. Therefore, in order to develop effective ergonomic and health intervention programs aimed at maintaining and improving employee health within a specific cultural and social context, it is necessary to identify, understand, and assess the intensity and relative importance of various stress factors and job satisfaction determinants, as well as to compare and apply their influence on work ability and individual well-being for each employee. All of this is intended to improve and enhance the balance between the level of effort required for the safe and high-quality performance of work tasks and the economic and social benefits—such as salary, status, and recognition—that a job provides as compensation for that effort, while also supporting family life.

## 5. Limitations

A potential limitation of this study is that the sample comprised slightly less than half of all employees working in emergency medical services in the Republic of Croatia. To consider the results fully representative at the national level, the required sample size would be 50% plus one participant more than the total population. Since the sample included somewhat less than 50% of all employees (43.3%), the findings provide an approximate picture of the situation at the national level. Nevertheless, it can be assumed that at least the majority of the conclusions apply to all employees in the Republic of Croatia. Furthermore, the sample was nearly three times larger than the minimum size required to achieve the objectives of this study.

Because the overall level of stress among employees was approximately two times lower than the reference value defining the threshold for stress, and because work ability scores measured by the Work Ability Index (WAI) were exceptionally high, a comprehensive multivariate regression model was not constructed. In addition, the study included a large number of dependent variables, including 20 open-ended questions. Therefore, potential predictors were examined individually using standard statistical tests rather than through a multivariate regression analysis.

The comparison of occupational stress levels and work ability scores between the control group and the case group did not achieve the objective of detecting the impact of the COVID-19 pandemic on stress and work ability. This was because the influence of stress associated with working in the Dispatch and Communication Unit (together with personal characteristics that may act as predictors of stress) was exceptionally strong and appeared to be greater than the impact of the pandemic itself. Nevertheless, the effect of the COVID-19 pandemic on stress levels among healthcare professionals is indisputable and has been extensively investigated in numerous scientific studies [49,50,51].

Future research should explore the reasons why employees choose to remain in or leave their positions, as the retention and recruitment of emergency medical service personnel is becoming a global challenge.

## 6. Conclusions

The results of the study conducted on slightly less than half of all employees confirm the stated hypothesis, namely that work-related stressors have a negative impact on the work ability of healthcare professionals in emergency medical intervention teams in the Republic of Croatia during the COVID-19 pandemic.

- Employees, on average, report lower levels of stress than the reference value, both for overall stress and for each individual stress factor examined.
- The stress scale related to the factor public criticism and lawsuits received the highest average score, although it remained significantly below the reference value.
- Employees, on average, rate their work ability as very good according to the WAI method.
- There is a weak negative correlation between all individual stress factors and work ability scores; in other words, increased stress has a relatively weak but statistically significant negative effect on employees’ work ability.
- Male respondents show higher work ability and lower overall perceived stress, while middle-aged and older respondents experience higher stress levels, and work ability decreases with increasing age.
- The lowest average stress levels across all measured factors were observed among respondents with the highest level of education; however, no association was found between education level and work ability assessed by the WAI method.
- Respondents in the control group report higher stress across all factors and lower work ability; however, they are also significantly older, have a lower level of education, and are more often female.
- Potential predictors of higher stress levels include: Female gender, middle or older age, working in a dispatch/communication unit, performing tasks not aligned with educational level, longer tenure in the current position, and total work experience.
- Potential predictors of lower stress levels include: Higher job satisfaction, a very high level of education, employee ambition, and the perception of doing a desired job.
- Potential predictors of lower work ability include: Female gender, longer tenure in the current position and total work experience, and potentially hazardous working conditions.
- Potential predictors of higher work ability include: employee ambition reflected in studying while working, job challenge, and the perception that it is the desired profession.
- Sick leave is also a potential predictor of higher stress levels and lower work ability, as employees who did not take sick leave show significantly lower stress levels and significantly higher work ability scores.

These results indicate that the average employee is not under significant stress and has very good work ability, and that higher job satisfaction and greater motivation are potential predictors of lower perceived stress and higher levels of work ability.

Further research is needed, including: longitudinal follow-up to determine changes in work ability (WAI), multivariate regression analysis adjusting for age, gender, and education to isolate the effect of the work environment, research focused on organizational and psychosocial factors that may contribute to the preservation of work ability and lower levels of occupational stress among emergency medical workers, and prospective studies to define objective criteria for the “adaptation phase” of employment in emergency medical services.

## Figures and Tables

**Table 1:** Demographic Characteristics of the Participants.

		Number (%) of Participants	*p*
Sex (*n* = 839)	Male	433 (51.6)	0.51
	Female	406 (48.4)	
Age (*n* = 838)	18–25	135 (16.1)	<0.001
	26–35	346 (41.3)	
	36–45	146 (17.4)	
	46–55	83 (9.9)	
	>55	128 (15.3)	
Highest level of education (*n* = 836)	Secondary education	443 (53.0)	<0.001
	Undergraduate professional/university degree	133 (15.9)	
	Graduate professional/university degree	246 (29.4)	
	Master of Science (MSc) or Doctor of Science (PhD)	14 (1.7)	

**Table 2:** Work-related characteristics of the respondents.

		Number (%) of Respondents	*p*
Occupation and job tasks (*n* = 839)	Physician	219 (26.1)	<0.001
	Nurse/Medical technician	437 (52.1)	
	Bachelor (undergraduate degree)	123 (14.7)	
	Master of Nursing/BSc in Med. Tech	29 (3.5)	
	Other	31 (3.7)	
Are the jobs aligned with level of education (*n* = 840)	Yes	792 (94.3)	<0.001
	No	48 (5.7)	
Are you currently studying while working (*n* = 833)	Yes, in my profession	143 (17.1)	<0.001
	Yes, but not in my profession	10 (1.2)	
	No, but I am considering it	185 (22.3)	
	No	495 (59.4)	
Are you employed on a permanent contract (*n* = 838)	Yes	729 (87.0)	<0.001
	No	109 (13.0)	
Workplace *	Team I	553 (65.8)	<0.001
	Team II	203 (24.2)	<0.001
	Dispatch unit	130 (15.5)	<0.001
	Medical transport	94 (11.2)	<0.001
	Other	19 (2.3)	<0.001
Length of service at current workplace (*n* = 834)	Up to 1 year	121 (14.5)	<0.001
	Between 1 and 5 years	254 (30.5)	
	Between 6 and 15 years	264 (31.7)	
	Between 16 and 30 years	141 (16.9)	
	More than 30 years	54 (6.5)	

* some employees, occasionally worked in both Team I and Team II and mentioned both jobs.

**Table 3:** Data Related to Job Satisfaction.

		Number (%) of Respondents	*p*
Job satisfaction (*n* = 836)	Very satisfied	224 (26.8)	<0.001
	Quite satisfied	356 (42.6)	
	Partially satisfied	219 (26.2)	
	Quite dissatisfied	29 (3.5)	
	Very dissatisfied	8 (1.0)	
How do you perceive your professional life overall? (*n* = 835)	Challenging, interesting, and meaningful	578 (69.2)	<0.001
	I don’t know, I work for the salary	149 (17.8)	
	As an obligation	58 (6.9)	
	As a coercion	3 (0.4)	
	Increasingly unpleasant over time	47 (5.6)	
Are your workplace conditions hazardous to your health? (*n* = 834)	Yes, they are hazardous	196 (23.5)	<0.001
	Mostly not, though unpredictable risks exist	516 (61.8)	
	Mostly not, though predictable risks exist	78 (9.3)	
	No, not at all	44 (5.3)	
Have work-related events caused your sick leave? (*n* = 839)	I did not take sick leave	372 (44.3)	<0.001
	Work often caused my sick leave	17 (2.0)	
	Work occasionally caused my sick leave	220 (26.2)	
	Work did not cause my sick leave	230 (27.4)	
Most frequent cause of sick leave in the last 3–6 months (*n* = 834)	I did not take sick leave	543 (65.1)	<0.001
	Family member illness	30 (3.6)	
	Own illness	218 (26.1)	
	Work-related injuries	43 (5.2)	
Is your current job also your desired job? (*n* = 838)	No, I work out of necessity but seek a better job	26 (3.1)	<0.001
	Mostly not, but I try to adapt myself/job to match my preferences	65 (7.8)	
	I don’t know, I work for the salary only	51 (6.1)	
	Close to what I want, but I would change if a better opportunity arises	302 (36.0)	
	This job is exactly what and how I want to work	394 (47.0)	
In the last 3–6 months, have you felt exposed to health-related stress at work? (*n* = 837)	Not at all	115 (13.7)	<0.001
	Slightly	210 (25.1)	
	Moderately	303 (36.2)	
	Quite strongly	154 (18.4)	
	Very strongly	55 (6.6)	

**Table 4:** Correlations between WAI and each stress factor.

(*n* = 792)	Stress Factors	rho	95% CI	*p*
Work Ability (assessed by WAI method) and Occupational Stress Factors	Organizational structure and financial issues	−0.33	−0.39 to −0.26	<0.001
	Public criticism and legal pressure (lawsuits)	−0.35	−0.41 to −0.29	<0.001
	Exposure to hazards and harmful working conditions	−0.21	−0.28 to −0.14	<0.001
	Workplace conflicts and communication problems	−0.26	−0.32 to −0.19	<0.001
	Shift work (work schedule demands)	−0.35	−0.41 to −0.29	<0.001
	Professional and intellectual demands	−0.30	−0.37 to −0.24	<0.001
	Overall perceived stress level	−0.37	−0.43 to −0.31	<0.001

Spearman rank correlation test.

**Table 5:** Comparison of Stress Factor Scores and Work Ability Index (WAI) between Male and Female Respondents.

	Median (Interquartile Range)		*p*
Stress Factors (*n* = 791)			
	Men/*n* = 410	Women/*n* = 381	
Organizational structure and financial issues	32.5 (17.5–50.0)	32.5 (19.4–47.5)	0.86
Public criticism and legal pressure (lawsuits)	39.3 (21.4–53.6)	46.4 (28.6–64.3)	<0.001
Exposure to hazards and harmful working conditions	20.8 (8.3–37.5)	20.8 (8.3–33.3)	0.33
Workplace conflicts and communication problems	18.8 (0.0–37.5)	25.0 (6.3–43.8)	0.03
Shift work (work schedule demands)	18.8 (0.0–43.8)	25.0 (6.3–50.0)	0.003
Professional and intellectual demands	20.8 (4.2–37.5)	25.0 (8.3–37.5)	0.08
Overall perceived stress level	27.0 (14.9–41.9)	31.1 (19.6–43.9)	0.03
Work Ability (*n* = 839)			
	Men/*n* = 433	Women/*n* = 406	
WAI	43.0 (38.0–46.0)	41.0 (37.0–45.0)	0.002

**Table 6:** Potential differences in work ability related to respondents’ workplace data.

		Median (Interquartile Range)	*p*
Occupation and job tasks (*n* = 838)	Physician	42.0 (39.0–45.0)	0.15
	Nurse/Medical technician	41.0 (36.0–45.0)	
	Bachelor (undergraduate degree)	42.0 (38.0–46.0)	
	Master of Nursing/BSc in Med. Tech	43.0 (37.0–47.0)	
Are the jobs aligned with level of education (*n* = 839)	Yes	42.0 (37.0–46.0)	0.25
	No	43.5 (39.0–45.5)	
Are you currently studying while working (*n* = 833)	Yes, in my profession	43.0 (40.0–47.0)	<0.001
	Yes, but not in my profession	42.0 (30.0–47.0)	
	No, but I am considering it	43.0 (38.8–46.0)	
	No	41.0 (37.0–45.0)	
Are you employed on a permanent contract (*n* = 837)	Yes	42.0 (37.0–45.0)	<0.001
	No	44.0 (40.0–47.0)	
Length of service at current workplace (*n* = 834)	Up to 1 year	43.0 (40.0–47.0)	<0.001
	Between 1 and 5 years	43.0 (40.0–47.0)	
	Between 6 and 15 years	42.0 (37.0–46.0)	
	Between 16 and 30 years	39.0 (35.0–44.0)	
	More than 30 years	37.0 (33.0–42.0)	
Overtime Work (*n* = 840)	Yes	42.0 (37.0–46.0)	0.51
	No	42.0 (37.8–46.0)	

**Table 7:** Comparison of Stress Factor Scores and Work Ability Index (WAI) between Respondents and the Control Group.

	Median (Interquartile Range)		*p*
Stress Factors (*n* = 792)			
	Respondents/*n* = 675	Control/*n* = 117	
Organizational structure and financial issues	30.0 (17.5–47.5)	40.0 (25.0–55.0)	<0.001
Public criticism and legal pressure (lawsuits)	39.3 (22.3–57.1)	57.1 (34.8–71.4)	<0.001
Exposure to hazards and harmful working conditions	20.8 (8.3–37.5)	16.7 (8.3–33.3)	0.04
Workplace conflicts and communication problems	18.8 (0.0–37.5)	25.0 (12.5–45.3)	0.005
Shift work (work schedule demands)	25.0 (0.0–43.8)	37.5 (17.2–50.0)	<0.001
Professional and intellectual demands	20.8 (5.2–37.5)	25.0 (12.5–45.8)	0.004
Overall perceived stress level	27.7 (16.2–41.9)	34.5 (23.5–47.6)	<0.001
Ability Index (*n* = 840)			
	Respondents/*n* = 710	Control/*n* = 130	
WAI	43.0 (38.0–46.0)	38.0 (33.0–42.0)	<0.001

**Table 8:** Potential Differences in Work Ability in Relation to Job Satisfaction.

		Median (Interquartile Range)	*p*
Job Satisfaction (*n* = 836)	Very satisfied	45.0 (41.0–48.0)	<0.001
	Quite satisfied	43.0 (39.0–45.0)	
	Moderately satisfied	39.0 (36.0–43.0)	
	Quite dissatisfied	40.0 (30.8–42.8)	
	Very dissatisfied	37.5 (32.5–43.5)	
How do you predominantly perceive your work life? (*n* = 830)	Challenging, interesting, and meaningful	43.0 (39.0–46.3)	<0.001
	I don’t know, I work for a salary	41.0 (37.0–45.0)	
	As an obligation	37.0 (34.0–41.3)	
	Increasingly aversive over time	37.0 (31.0–41.0)	
Do workplace conditions pose a risk to your health? (*n* = 834)	Yes, they are harmful	40.0 (35.0–45.0)	<0.001
	Mostly not, although there are possible unpredictable circumstances	43.0 (38.0–46.0)	
	Mostly not, although there are possible predictable circumstances	42.0 (37.0–46.0)	
	No, not at all	43.0 (40.0–47.0)	
Have work-related events caused you to take sick leave? (*n* = 839)	I have not taken sick leave	45.0 (41.0–47.0)	<0.001
	Work has often been the cause of sick leave	35.0 (28.8–42.0)	
	Work has occasionally been the cause of sick leave	38.0 (34.0–42.0)	
	Work has not been the cause of sick leave	41.0 (38.0–45.0)	
What has been the most common reason for your sick leave in the past 3–6 months? (*n* = 834)	I have not taken sick leave	43.0 (39.0–47.0)	<0.001
	Illness of a family member	40.5 (37.0–45.0)	
	Personal illness	39.0 (35.0–43.0)	
	Work-related injuries	37.0 (32.0–41.0)	
Is your current job the job you want to do? (*n* = 838)	No, I do this job out of necessity but I am looking for a better one	41.5 (38.0–43.0)	<0.001
	Not entirely, but I am working on adapting myself/the job to better match my preferences	39.0 (32.0–42.0)	
	I don’t know, I work for a salary and that is what matters	37.0 (34.3–43.8)	
	It is close to what I want to do, but I would change it if a better opportunity arose	41.0 (37.0–45.0)	
	This job is exactly what I want to do	44.0 (39.0–47.0)	
In the past 3–6 months, have you felt exposed to health-related strain at work? (*n* = 837)	Not at all	45.0 (41.3–48.0)	<0.001
	Slightly	44.0 (39.0–46.0)	
	Moderately	42.0 (38.0–45.0)	
	Quite strongly	38.0 (34.0–42.0)	
	Very strongly	40.0 (35.0–44.8)	

Kruskal–Wallis test.

## Data Availability

The original data presented in the study are openly available in [Zenodo] at [https://doi.org/10.5281/zenodo.19667128].
